# Boosting the Accuracy of Commercial Real Estate Appraisals: An Interpretable Machine Learning Approach

**DOI:** 10.1007/s11146-023-09944-1

**Published:** 2023-03-22

**Authors:** Juergen Deppner, Benedict von Ahlefeldt-Dehn, Eli Beracha, Wolfgang Schaefers

**Affiliations:** 1 University of Regensburg, IRE|BS International Real Estate Business School, Regensburg, Germany; 2 Florida International University, Hollo School of Real Estate, FL Miami, USA

**Keywords:** Commercial real estate, Appraisal, Interpretable machine learning

## Abstract

In this article, we examine the accuracy and bias of market valuations in the U.S. commercial real estate sector using properties included in the NCREIF Property Index (NPI) between 1997 and 2021 and assess the potential of machine learning algorithms (i.e., boosting trees) to shrink the deviations between market values and subsequent transaction prices. Under consideration of 50 covariates, we find that these deviations exhibit structured variation that boosting trees can capture and further explain, thereby increasing appraisal accuracy and eliminating structural bias. The understanding of the models is greatest for apartments and industrial properties, followed by office and retail buildings. This study is the first in the literature to extend the application of machine learning in the context of property pricing and valuation from residential use types and commercial multifamily to office, retail, and industrial assets. In addition, this article contributes to the existing literature by providing an indication of the room for improvement in state-of-the-art valuation practices in the U.S. commercial real estate sector that can be exploited by using the guidance of supervised machine learning methods. The contributions of this study are, thus, timely and important to many parties in the real estate sector, including authorities, banks, insurers and pension and sovereign wealth funds.

## Introduction

Both institutional and private investors aim to diversify their portfolios with real estate. A significant share of this is accounted for by investments in commercial real estate sectors, which amount to around $32 trillion globally. The heterogeneity of commercial real estate contributes well to diversification, but it is also accompanied by characteristics such as illiquidity, opacity and unwieldiness that make it difficult to thoroughly understand market dynamics. Consequently, the valuation of commercial properties involves a great deal of effort that justifies an appraisal industry worth billions of dollars. Studies have repeatedly demonstrated that commercial property appraisals do not always adequately represent market dynamics and can differ significantly from actual sales prices (e.g., Cole et al., 1986; Webb, 1994; Fisher et al., 1999; Matysiak & Wang, 1995; Edelstein & Quan, 2006; Cannon & Cole, 2011). Despite the increasing complexity of pricing processes and more rapidly changing markets, the principal methods used by the valuation industry have largely remained unchanged for the past decades. However, this is slowly changing with an increasing availability of data and the emergence of artificial intelligence fostering the use of innovative technologies in the real estate sector.

In recent years, machine learning algorithms have been increasingly considered as a suitable method for the estimation of house prices and rents, with a large corpus of literature pointing to their high accuracy in the residential sector (e.g., Mullainathan & Spiess, 2017; Mayer et al., 2019; Bogin & Shui, 2020; Hong et al., 2020; Pace & Hayunga, 2020; Lorenz et al., 2022; and Deppner & Cajias, 2022). In the commercial sector, on the other hand, the scope of analysis has thus far been limited to multifamily assets and shows inconsistent results in terms of estimation accuracy (Kok et al., 2017). One prerequisite for machine learning methods to provide accurate and reliable property value estimates is the availability of substantial amounts of data with uniform property characteristics. While these criteria are largely met for residential real estate where property characteristics are considered relatively homogeneous, and data is widely accessible on multiple listing services, the nature of commercial real estate is more complex and heterogenous, and infrequent transactions and market opaqueness continue to hinder data availability. Despite the enormous potential for the sector, this poses a challenge for the application of data-driven valuation methods in commercial real estate and raises the question to what extent machine learning algorithms can provide significant improvement to the industry’s state-of-the-art appraisal practices. To the best of our knowledge, there is no research in the current literature that investigates the usefulness of machine learning algorithms for the valuation of commercial properties other than multifamily buildings (see Kok et al., 2017).

This article contributes to this field using 24 years of property-level transaction data of commercial real estate from the NCREIF Property Index (NPI) provided by the National Council of Real Estate Investment Fiduciaries (NCREIF). In a first step, we investigate the deviation between actual sales prices observed in the market and the appraised values before sale to assess the accuracy and bias associated with state-of-the-art valuation methods that were last examined by Cannon and Cole (2011). Given the findings of inaccuracy and structural bias of appraisals that the literature has reported over the past decades, we hypothesize that the observed deviations between sales prices and appraisal values exhibit structured information content that machine learning models can exploit to further explain and shrink these residuals, thereby providing a superior ex post understanding of market dynamics. This is examined using a tree-based boosting algorithm, measuring how much of the variation in the residuals can be explained. While Pace and Hayunga (2020) follow a similar approach to benchmark machine learning methods against spatial hedonic tools in a residential context, no research empirically quantifies the potential of complementing traditional appraisal methods with data-driven machine learning techniques, neither in residential nor commercial sectors. Lastly, we apply model-agnostic permutation feature importance to reveal where improvements originate and point to price determinants that are not adequately reflected in current appraisal methods.

From a practical point of view, the application of machine learning can add to an enhanced ex ante understanding of pricing processes that may support valuers in the industry and contribute to more dependable valuations in the future. By illustrating the potential and pointing to the shortcomings of these methods, we aim to provide guidance, stimulate the critical discussion, and motivate further research on machine learning approaches in the context of commercial real estate valuation.

## Related Literature

The estimation of market values is the primary concern of most real estate appraisal assignments. According to federal financial institutions in the U.S., the market value is defined as:

“*[…] the most probable price which a property should bring in a competitive and open market under all conditions requisite to a fair sale, the buyer and seller each acting prudently and knowledgeably, and assuming the price is not affected by undue stimulus*”^1^ (Real Estate Lending and Appraisals, 2022).

However, the accurate and timely estimation of commercial property prices is a complex task, as direct real estate markets are characterized by high heterogeneity, illiquidity, and information asymmetries that are accompanied by high search and transaction costs. Over the past decades, many methods have been developed and refined to arrive at the most probable transaction price of a property in the market. Pagourtzi et al. (2003) distinguish between traditional (i.e., manual) and advanced (i.e., statistical) valuation approaches.

### Traditional Valuation Methods

Traditional valuation models are characterized by a procedural approach (Mullainathan & Spiess, 2017) that follows pre-defined economic rules. These procedures can be thought of as ‘prediction rules’ used to obtain appraised values of commercial real estate. The most common procedures in current appraisal practices are the *income approach*, the *sales-comparison approach* and the *cost approach* as described by Fisher and Martin (2004) and Mooya (2016).

As the industry´s preferred approach to commercial property valuation, the *income approach* is based on the idea that the value of a property depends on the present value of its future cash flows, and is thus determined by two main factors: the net operating income and the capitalization rate. The latter incorporates all risks and upside potentials of the income-producing property. However, the correct assessment of the capitalization rate is not straightforward and depends on many assumptions. Hence, comparable transactions of similar properties observed in the market are often used as a point of reference. This is known as the *sales-comparison approach* and is based on the rationale that the value of a property should equal the value of a similar property with the same characteristics. Mooya (2016) finds this approach to be the most valid indicator of market conditions as new market valuations are based on recently transacted properties. However, comparable sales are scarce or outdated in very illiquid property sectors and markets. In such cases, the *cost approach* can be used following the principle that an informed investor would pay no more than for the substitute building as this would constitute an arbitrage opportunity. The market value of a property is thus derived from the cost of constructing a similar property including the land value and adjusting for physical and functional depreciation.

All these procedures have an economic justification and have served the industry well for decades; however, as prediction rules, they also suffer from certain limitations. For instance, the determination of the capitalization rate is subject to the discretionary scope and the assumptions (i.e., the assessment of risks and upside potentials, e.g., growth hypothesis versus risk hypothesis for vacant space in Beracha et al., 2019) of the individual executing them to arrive at a market value. In turn, capitalization rates derived from comparable sales may capture recent market dynamics but are inherently backwards looking such that appraisals may significantly lag. Furthermore, the availability of similar properties that have been sold recently is a limiting factor due to infrequent transactions and high heterogeneity. This requires adjustments, which again depend on subjective opinions of value, resulting in imprecise estimations. On the other hand, the cost approach can indicate a property’s substitute value, but also allows a lot of room for subjectivity given the uniqueness of each property and the numerous assumptions to be made for adjustments and depreciation. Pagourtzi et al. (2003) note that “[…] price will be determined not by cost, but by the supply and demand characteristics of the occupational market” in case of scarcity, which is a typical characteristic of many real estate markets due to geographic constraints and building regulations. In addition, Matysiak and Wang (1995) raise the hypothesis that not all available data is considered at the time of valuation. While each of the approaches mentioned above is limited to a certain set of information, market intransparency may furthermore impose restrictions to the data that is available to individual appraisers.

Cole et al. (1986) are the first in the literature to document the differences between real estate appraisals and sales prices in the U.S. commercial real estate market. The authors examine properties sold out of the NCREIF Property Index (NPI) between 1978 and 1984 and find a mean absolute percentage difference of around 9% in that period of rising markets. In a similar study, Webb (1994) extends the sample of Cole et al. (1986) by updating the period from 1978 to 1992, thereby covering different price regimes of rising, stagnating, and falling markets. The author finds that the highest deviations occur during rising markets averaging 13%, declining to 10% during flat markets and 7% during falling markets. Fisher et al. (1999) update the studies of Cole et al. (1986) and Webb (1994) on the reliability of commercial real estate appraisals in the U.S. and show that from 1978 to 1998, manual appraisals of NPI properties across multiple asset types deviate on average between 9% and 12.5% from actual sales prices. This is in line with the findings of Cannon and Cole (2011) who analyzed NPI sales data from 1984 to 2009 and observed deviations ranging between 11% and 13.5% over the entire sample period for the different asset sectors. The authors find appraisals to consistently lag actual sales prices, falling short of sales prices in bullish markets and remaining in excess of sales prices in bearish markets. With respect to mean percentage errors, the findings of Cannon and Cole (2011) confirm the hypothesis of Matysiak and Wang (1995), suggesting that appraisal errors do not solely arise due to the time differences but also due to a systematic valuation bias. Kok et al. (2017) take another look at appraisal errors in commercial real estate markets and propose the use of advanced statistical techniques to reduce the deviations found in the previous studies.

### Advanced Valuation Methods

With an increasing data availability in real estate markets and the development of econometric and statistical techniques, researchers have started to tackle existing tasks empirically instead of procedurally (Mullainathan & Spiess, 2017). While a wide range of empirical methods exists in the current literature, we focus on the most discussed approaches for property valuation, that is *hedonic pricing* and *machine learning*.

The *hedonic pricing* model dates to Rosen (1974) who defines the value of a heterogenous good as the sum of the implicit prices of its objectively measurable characteristics. The most common econometric approach used to derive such implicit prices is multiple linear regression or extensions thereof. In commercial real estate markets, hedonic pricing models have been applied to disentangle price formation processes from an econometric point of view (e.g., Clapp, 1980; Brennan et al., 1984; Glascock et al., 1990; Mills, 1992; Malpezzi, 2002; Sirmans et al., 2005; Koppels and Soeter, 2006; Nappi-Choulet et al., 2007; Seo et al., 2019). Hedonic models have proven useful in understanding price determinants in real estate markets, but researchers have also pointed to the limitations of the underlying methods such as their imposed linearity and fixed parameters, which cannot be assumed to hold in reality (Dunse & Jones, 1998; Bourassa et al., 2010; Osland, 2010). Although these models are efficient in generating predictions and easy to interpret, their strong assumptions and need for manual specification carry the risk of bias, subjectivity, and inconsistency, which is to be eliminated in the first place.

In contrast to linear hedonic approaches, algorithmic *machine learning* models follow a purely data-driven approach and make use of stochastic rules to find the best possible model fit. Over the past decades, many algorithms such as artificial neural networks (Rumelhart et al., 1986), support vector regression (Smola & Schölkopf, 2004), and bagging and boosting algorithms (i.e., random forest regression by Breiman, 1996, 2001 and gradient tree boosting by Friedman, 2001) that are based on ensembles of regression trees (Breiman et al., 1984) have been developed and refined. These algorithms can autonomously learn non-linear relationships from the data without specifying them a-priori or making any implicit assumptions of the relationship between the property’s price and its features. This means that the models consider all available information at the time of valuation and identify complex relationships based on patterns in the data. Since the training process of machine learning algorithms is computationally expensive compared to traditional econometric models, it took until this decade for technological progress to enable sufficient computational capacity for the widespread application of such techniques.

In recent years, a large corpus of literature has demonstrated the potential of machine learning algorithms to accurately estimate prices and rents of houses and apartments in the residential sector. This includes studies by McCluskey et al. (2013) for artificial neural networks, Lam et al. (2009), Kontrimas and Verikas (2011), and Pai and Wang (2020) for support vector regression, Levantesi and Piscopo (2020) for random forest regression and van Wezel et al. (2005) and Sing et al. (2021) for gradient tree boosting algorithms. In many comparative studies that document the accuracy of a broader range of model alternatives, tree-based methods and, in particular boosting and bagging algorithms, have shown superiority over other methods (e.g., Zurada et al., 2011; Antipov & Pokryshevskaya, 2012; Mullainathan & Spiess, 2017; Baldominos et al., 2018; Hu et al., 2019; Mayer et al., 2019; Bogin & Shui, 2020; Pace & Hayunga, 2020; Cajias et al., 2021; Rico-Juan and Taltavull de La Paz, 2022; Lorenz et al., 2022; and Deppner & Cajias, 2022).

In academia and the industry, however, high demands are placed not only on accuracy and consistency, but also on reliability and comprehensibility of the models. Hence, machine learning methods have been criticized for lacking an economic justification and having a black-box character (Mayer et al., 2019; McCluskey et al., 2013). Valier (2020) argues that although data-driven machine learning models might produce equivalent or even better results than traditional methods, too much variability comes with the flexibility of these methods as they rely entirely on the input data and can change quickly. This makes them “[…] difficult to use for public policies, where the evaluation process must guarantee fairness of treatment for all the cases concerned and maintain the same efficiency over time,” as stated by Valier (2020). While Pérez-Rave et al. (2019) and Pace and Hayunga (2020) suggest to maintain interpretability by enhancing linear models with insights generated by machine learning techniques, Rico-Juan and Taltavull de La Paz (2022) and Lorenz et al. (2022) apply model-agnostic interpretation techniques that allow ex-post interpretability of the models to circumvent this problem.

Besides their sensitivity to changes in the data, the methods can quickly overfit the training sample if applied without the necessary prudence and may thus not represent the true relationship between the dependent variable and its regressors. This is especially problematic when training data is scarce. For this reason, machine learning algorithms require a reasonable number of observations of previous transactions and attributes that adequately describe the respective properties to provide dependable and stable estimations of property values. Hence, research in this field has largely focused on the residential sector, where properties are considered relatively homogeneous, and data availability has increased exponentially over the last years with the transition from offline real estate offers to online multiple listing services. In turn, the high heterogeneity and data scarcity in commercial real estate markets imposes challenges for the application of machine learning techniques. Kok et al. (2017) are the first in the literature to apply machine learning methods to estimate prices of commercial multifamily properties. The authors benchmark tree-based boosting and bagging algorithms against a linear hedonic model across different model specifications and find mixed results in terms of their accuracy. While two different types of boosting provide error reduction in all cases tested, the bagging algorithm does not offer any significant improvement and is even outperformed by the ordinary least squares estimator in one case. To the best of our knowledge, there is no research on the predictive performance of machine learning methods for other property types in commercial real estate.

Although institutionally held multifamily properties are of residential use, the study of Kok et al. (2017) indicates that previous findings of the accuracy of machine learning algorithms in the residential sector cannot be easily transferred to a commercial real estate context, given the known limitations of these techniques and the peculiarities of the sector as discussed earlier. This raises the question to which extent algorithmic approaches can learn market dynamics in commercial real estate to generate insights into pricing processes that go beyond the understanding achieved with traditional valuation approaches, thus providing potential improvement to the state-of-the-art.

## Data and Methodology

The principal dataset used for this study was provided by the National Council of Real Estate Investment Fiduciaries (NCREIF). It contains quarterly observations of all properties included in the NCREIF Property Index^2^ (NPI) on the asset level spanning 1Q 1978 through 1Q 2021. To be included in the NPI, a property must be.

i. an operating apartment, hotel, industrial, office, or retail property,
ii. acquired, at least in part, by tax-exempt institutional investors and held in a fiduciary environment,^3^
iii. accounted for in compliance with the NCREIF Market Value Accounting Policy,^4^
iv. appraised – either internally or externally – at a minimum every quarter.

A qualifying property is included in the NPI upon purchase and removed again upon sale. The database contains all quarter-observations over that property’s holding period, terminating with the sale quarter. For reasons of data scarcity in earlier years and in specific sectors, we limit the initial sample to 24 years from 1Q 1997 through 1Q 2021, including all asset sectors except for hotels. This is generally equivalent to the dataset in the study of Cannon and Cole (2011), with the time span shifted 12 years ahead.

### Data Pre-processing

We filter all properties that had been sold during that period, excluding partial sales and transfers of ownership. This constitutes a sample of 12,956 individual assets for which we observe the net sale prices, the corresponding appraisal values and a series of structural, physical, financial, and spatial attributes recorded quarterly.

After examining the most recent appraisal values of the sold properties from the quarter before the sale, we find that the appraised value equals the net sale price in 6,091 cases, which corresponds to 47% of the entire sample. This is consistent with Cannon and Cole (2011) and indicates that the sale price for those properties was determined at least three months before a pending transaction. Since this price was used as the market value instead of an independent appraisal, we are forced to use the appraisal values of the second quarter before the sale to represent the properties’ most recent market value. However, we still observe 587 properties where the market value equals the sale price and another 179 properties with missing data for that quarter, resulting in a reduced sample of 12,190 properties for which we have data on the sale prices and the market values. One possibility to account for the time lag between the appraisal date and the sale date is to roll back the sale prices as Cannon and Cole (2011) did for some properties in their sample. However, the authors find that overall, the unadjusted differences are, in fact, better measures of appraisal accuracy. This is no surprise as transaction prices are often determined three to six months before closing, known as due diligence lag. We subsequently do not adjust for the time lag between appraisal and sale date but control for moving markets in that period.

Missing and erroneous data points of the relevant variables are accounted for as follows. We remove observations with square footage and construction years reported as less than or equal to zero. Likewise, occupancy rates less than zero or higher than one were also regarded as erroneous data points. Furthermore, we omit observations with missing values for the square footage, the property subtype, the construction year, the occupancy rate, the appraisal type, the fund type, the metropolitan statistical area (MSA) code, the net operating income (NOI), and the capital expenditures (Capex), which represent the main explanatory variables collected from the raw, principal dataset. We further remove observations where the deviation between the sale price and the appraisal value two quarters before the sale is abnormally high, as this indicates a potential data error.^5^ We also remove extreme outliers in the sale price, the building area and the sale price per square foot by cropping the upper and lower tails of the distributions.^6^ After cleaning erroneous and missing data, the sample was reduced to 8,427 individual properties.

In addition, we enrich the initial data with a set of new variables. To better control for building quality, we calculate the building age as the difference between the year of sale and the construction date trimmed at 100 years^7^ and the cumulative sum of a property’s capital expenditures, that is the sum of all capital expenditures for building extensions and building improvements over the holding period.^8^ Since we observe that NOIs tend to fluctuate materially in the quarters before sale, we also calculate the mean of the properties’ annual NOIs over their holding period as a proxy for stabilized income. This measure incorporates different market cycles and is less prone to speculation, which may better capture a property’s intrinsic value.

As demonstrated repeatedly in the literature, the spatial dimension is an important driver of real estate prices. The dataset provides the location zones of a property on the ZIP code level. However, we cannot ensure enough observations for each ZIP code area in our sample, so we use the MSA level instead. That said, location dummies on the MSA level may capture global price differentials across space, but they are not adequate to efficiently reflect complex pricing behaviors driven by spatial considerations of buyers and sellers. To better assess appraisers’ understanding of space, we geocode our sample observations using the property addresses. With the Google Places API, we managed to geocode 93%^9^ of the addresses and retrieve the distances to relevant points of interest (POIs). This includes transport linkages and amenities that may produce spillover effects and thus cause positive or negative externalities to their neighborhood. For example, an office building might benefit from the proximity to a café, a gym or a laundry that serves white-collar workers, which translates into a location premium. Lastly, we omit MSA codes that include less than ten properties of the same asset class to counteract overfitting on the location dummies. Our final sample contains 7,133 individual properties^10^ that meet all the previously outlined criteria to be included in the study. Relative to the initial sample size this constitutes a heavy data loss, which again emphasizes the problem of data availability as mentioned earlier.^11^ Table 1 provides an overview of the number of observations across the sample period.

**Table 1:** Observations per Year

	All Types (N = 7,133)		Apartment (N = 1,904)		Industrial (N = 2,337)		Office (N = 2,056)		Retail (N = 836)	
Variable	n	Percent	n	Percent	n	Percent	n	Percent	n	Percent
Year										
… 1997	68	0.95%	17	0.89%	31	1.33%	9	0.44%	11	1.32%
… 1998	84	1.18%	12	0.63%	26	1.11%	31	1.51%	15	1.79%
… 1999	94	1.32%	18	0.95%	18	0.77%	31	1.51%	27	3.23%
… 2000	201	2.82%	51	2.68%	49	2.10%	74	3.60%	27	3.23%
… 2001	174	2.44%	53	2.78%	50	2.14%	42	2.04%	29	3.47%
… 2002	187	2.62%	49	2.57%	63	2.70%	51	2.48%	24	2.87%
… 2003	251	3.52%	60	3.15%	78	3.34%	80	3.89%	33	3.95%
… 2004	337	4.72%	74	3.89%	117	5.01%	107	5.20%	39	4.67%
… 2005	472	6.62%	109	5.72%	135	5.78%	132	6.42%	96	11.48%
… 2006	298	4.18%	75	3.94%	84	3.59%	115	5.59%	24	2.87%
… 2007	381	5.34%	91	4.78%	139	5.95%	124	6.03%	27	3.23%
… 2008	155	2.17%	42	2.21%	54	2.31%	53	2.58%	6	0.72%
… 2009	160	2.24%	57	2.99%	54	2.31%	40	1.95%	9	1.08%
… 2010	182	2.55%	66	3.47%	56	2.40%	40	1.95%	20	2.39%
… 2011	252	3.53%	68	3.57%	87	3.72%	50	2.43%	47	5.62%
… 2012	415	5.82%	112	5.88%	162	6.93%	100	4.86%	41	4.90%
… 2013	500	7.01%	149	7.83%	160	6.85%	122	5.93%	69	8.25%
… 2014	502	7.04%	112	5.88%	194	8.30%	137	6.66%	59	7.06%
… 2015	440	6.17%	130	6.83%	135	5.78%	126	6.13%	49	5.86%
… 2016	512	7.18%	154	8.09%	162	6.93%	146	7.10%	50	5.98%
… 2017	422	5.92%	126	6.62%	136	5.82%	123	5.98%	37	4.43%
… 2018	345	4.84%	119	6.25%	71	3.04%	140	6.81%	15	1.79%
… 2019	427	5.99%	90	4.73%	181	7.74%	110	5.35%	46	5.50%
… 2020	209	2.93%	60	3.15%	57	2.44%	59	2.87%	33	3.95%
… 2021	65	0.91%	10	0.53%	38	1.63%	14	0.68%	3	0.36%

This table presents the distribution of observations across the sample period from 1Q 1997 through 1Q 2021

We further follow Cannon and Cole (2011) in collecting macroeconomic data to control for structural differences in property prices across time. That includes the four-quarter percentage change in employment at the county-level sourced from the U.S. Bureau of Labor Statistics, the four-quarter percentage change in the gross domestic product (GDP) and the ten-year government bond yield sourced from the database of the Federal Reserve Bank of St. Louis, and the four-quarter percentage change in construction costs by region sourced from the U.S. Census Bureau. We further collect quarterly NPI data by property type, that is, the quarterly change in market value cap rates, vacancy rates, NOI growth rates and the quarterly number of sales of NPI properties. While all these variables capture the period between the sale date and the first quarter before sale, we also provide the lags of all macroeconomic and NPI index data for the period between the first and the second quarter prior to sale to control for the time lag between the appraisal and the sales date.

### Appraisal Error

NCREIF follows the definition of market value as stated in the "Related Literature" section and adopted by the Appraisal Foundation as well as by the Appraisal Institute. According to this definition, the market value of a property represents the best estimate of a transaction price in the current market. Consequently, we assess the manual appraisals as predictions of sales prices by examining the mean absolute percentage error (MAPE) and the mean percentage error (MPE) as calculated in Eqs. (1) and (2), respectively.

1 $$MAPE=\frac1n\sum\limits_{i=1}^n\left|\frac{{Sale\;Price}_{i,t0}-{Appraised\;Value}_{i,t-2}}{{Appraised\;Value}_{i,t-2}}\right|$$

2 $$MPE=\frac1n\sum\limits_{i=1}^n\frac{{Sale\;Price}_{i,t0}-{Appraised\;Value}_{i,t-2}}{{Appraised\;Value}_{i,t-2}}$$

The MAPE is used as a measure of accuracy, whereas the MPE can be understood as a measure of biasedness. That is, the appraised value is considered an unbiased predictor of sales prices, if the MPE is not significantly different from zero. This is examined using t-test statistics.

The vector of appraisal errors *Y* used as the dependent variable in our models is calculated as the difference between the vector of the log sale price per square foot (*SP*) and the vector of the log appraisal (market) value per square foot (*MV*). This is stated in Eq. (3), which corresponds to the log of the percentage appraisal error, however, keeping the signs.

3 $$\begin{array}{c}Y=\lbrack SP-MV\rbrack\\SP=\mathit{log}\left(\frac{{Sale\;Price}_{t0}}{SqFt}\right)\\MV=\mathit{log}\left(\frac{{Appraised\;Value}_{t-2}}{SqFt}\right)\end{array}$$

**Fig. 1:**
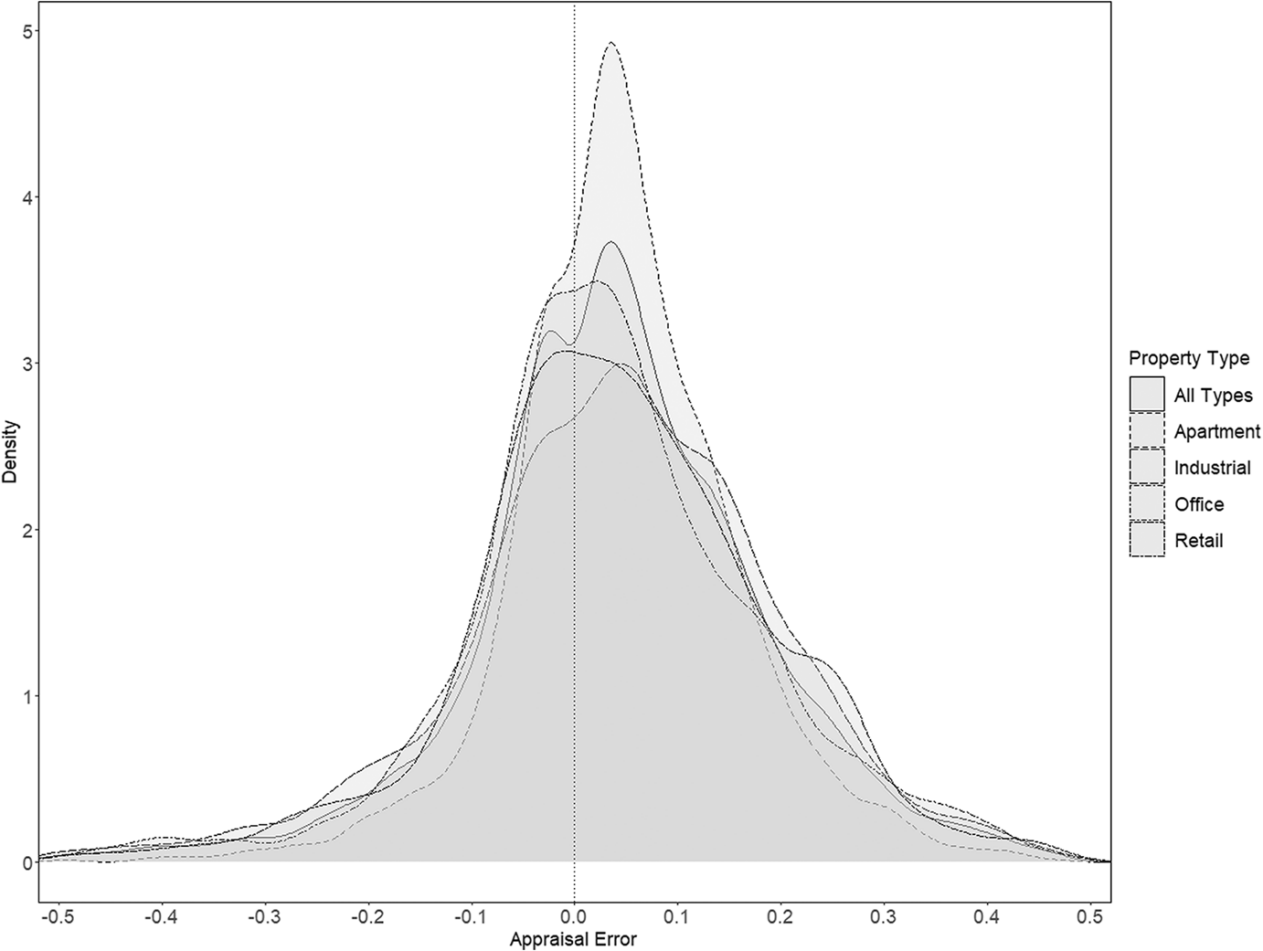
Distribution of Appraisal Errors. Notes: The density plot shows the distribution of the raw residuals (appraisal errors) for all property types and for each property type individually. The dotted horizontal line marks the null point on the x-axis

Figure 1 depicts the distribution of the dependent variable for the different property types. We expect systematic differences between appraisal errors of the four property types, so we conduct an analysis of variance (ANOVA) test with the null hypothesis that there is no significant difference in the sample means of the respective groupings. The ANOVA test rejects the null at the 1% level of significance, indicating systematic differences in the sample distributions of the four asset sectors.

### Explanatory Variables

Matysiak and Wang (1995) state that appraisal errors are generally rooted in two components. First, markets can change between the appraisal date and the sale date and second, a pure valuation error (i.e., bias) can be incorporated. The latter could be ruled out if the mean percentage error approaches zero, as positive and negative deviations should cancel out. If this is not the case, appraisal errors are unlikely to be entirely random, implying that some information content is left to be explained. To capture the two components from which deviations between appraised values and sales prices originate according to Matysiak and Wang (1995), we include a wide range of explanatory variables in our models.

The first component *a* refers to the time difference between the appraisal and transaction dates. That is, an appraisal error occurs due to a changing market environment during that period. To control for moving markets, we include the market indicators ${M}_{t0}$ and ${M}_{t-1}$ from the NPI data (i.e., the quarterly change in market value cap rates, vacancy rates, NOI growth rates and the quarterly number of sales of NPI properties as a proxy for market liquidity) for both quarters before sale as well as the continuous transaction year as temporal indicator *T*. However, a change in the value of a property could also result from a change in the property fundamentals. Although cash flows from the quarters before sale are backward-looking, and property values are inherently determined by future cash flows that can be estimated with existing lease contracts and maintenance plans, we control for the occurrence of unexpected events (such as rent defaults or repairs) by including the cash flows ${C}_{t0}$ and ${C}_{t-1}$ (that is the NOI and Capex) for both quarters before sale. The first component *a* of regressors can be specified in matrix notation as in Eq. (4).

4 $$X_a=\lbrack M_{t0}\;M_{t-1}T\;C_{t0}\;C_{t-1}\rbrack$$

The second component *b* refers to the pure valuation bias and can have various causes such as subjective opinions of value, varying risk appetite and assumptions of funds and individual appraisers or appraisal smoothing. To capture these effects, we include several structural (*S*), physical (*P*), financial (*F*), and locational (i.e., spatial) (*L*) property characteristics as well as economic (*E*) indicators for both quarters before sale, as specified in Eq. (5). This includes the fund type and the type of appraisal and the building occupancy for *S*, the property subtype, the building area, and the building age for *P*, the stabilized NOI and the cumulative sum of Capex for *F*, the MSA, latitude, longitude and distances to 18 POIs for *L*, as well as the four-quarter percentage change in employment on the county-level, the four-quarter percentage change in the GDP, the 10-year government bond yield, and the four-quarter percentage change in construction costs by region in both quarters prior to sale, corresponding to ${E}_{t0}$ and ${E}_{t-1}$ respectively. The covariates included in component *b* can thus be summarized as in Eq. (5).

5 $$X_b=\lbrack S\;P\;F\;L\;E_{t0}\;E_{t-1}\rbrack$$

Our models incorporate 50 explanatory variables reflecting the main information used in the traditional appraisal methods discussed in the "Traditional Valuation Methods" section (i.e., income approach, sales comparison approach, cost approach). The input–output relationship is summarized in Eq. (6).

6 $$Y\sim\lbrack X_a\;X_b\rbrack$$

Table 2 provides a summary statistic of all numerical regressors, and Table 3 presents the distributions of the categorical features. It should be mentioned that, aside from the components ${X}_{a}$ and ${X}_{b}$ following Matysiak and Wang (1995), appraisal values remain estimates and can rationally deviate from transaction prices for several reasons that are specific to the buyer or seller in the bargaining process and thus not foreseeable. However, we do not expect anything systematic in deviations of this kind, so we do not consider these random effects further.

**Table 2:** Descriptive Statistics of Numerical Variables

			All Types (N = 7,133)				
	Variable	Unit	Mean	Median	Sd	Min	Max
[T]	Year	[Years]	2010.74	2012.00	6.18	1997.00	2021.00
[P]	SqFt	[k]	273.43	203.29	283.02	2.25	5,995.50
	Building Age	[Years]	22.68	19.00	16.23	0.00	100.00
[S]	Occupancy	[%]	0.91	0.95	0.15	0.00	1.00
[F]	CapEx Cumulative	[$/SqFt]	14.45	3.36	188.43	0.00	15,518.44
	Stabilized NOI	[$/SqFt]	8.21	6.70	5.75	0.01	45.54
[C_t0_]	CapEx	[$/SqFt]	0.72	0.04	2.86	0.00	77.85
	NOI	[$/SqFt]	1.32	0.92	2.07	-53.10	46.73
[C_t-1_]	CapEx (lag)	[$/SqFt]	0.76	0.16	2.45	0.00	58.59
	NOI (lag)	[$/SqFt]	2.35	1.83	2.16	-8.55	31.79
[L]	Longitude	[°]	-95.46	-93.27	17.19	-122.93	-70.49
	Latitude	[°]	36.69	37.38	5.21	25.60	47.94
	Bank	[km]	0.75	0.52	0.77	0.00	6.49
	Bar	[km]	0.73	0.51	0.69	0.00	5.86
	Cafe	[km]	0.59	0.42	0.59	0.00	5.18
	Convenience Store	[km]	0.66	0.53	0.54	0.00	5.91
	Department Store	[km]	1.92	1.39	1.87	0.00	8.68
	Doctor	[km]	0.37	0.23	0.44	0.00	6.65
	Gas Station	[km]	0.73	0.61	0.54	0.00	5.59
	Gym	[km]	0.62	0.43	0.62	0.00	5.85
	Laundry	[km]	0.71	0.53	0.65	0.00	5.92
	Lawyer	[km]	0.58	0.35	0.71	0.00	6.28
	Park	[km]	0.70	0.57	0.56	0.00	6.31
	Parking	[km]	0.82	0.56	0.88	0.00	8.48
	Pharmacy	[km]	0.71	0.51	0.68	0.00	6.48
	Restaurant	[km]	0.36	0.24	0.39	0.00	3.78
	School	[km]	0.43	0.32	0.40	0.00	4.20
	Shopping mall	[km]	0.87	0.63	0.84	0.00	7.19
	Supermarket	[km]	1.37	1.02	1.30	0.00	8.66
	Public Transport	[km]	2.02	1.33	2.15	0.00	8.68
[E_t0_]	GDP yoy	[%]	0.02	0.02	0.01	-0.09	0.05
	Bond Yield	[%]	0.03	0.03	0.01	0.01	0.07
	Construction Cost yoy	[%]	0.04	0.04	0.04	-0.10	0.20
	Employment yoy	[%]	0.02	0.02	0.03	-0.18	0.27
[E_t-1_]	GDP yoy (lag)	[%]	0.02	0.02	0.02	-0.09	0.05
	Bond Yield (lag)	[%]	0.03	0.03	0.01	0.01	0.07
	Construction Cost yoy (lag)	[%]	0.04	0.04	0.04	-0.10	0.13
	Employment yoy (lag)	[%]	0.02	0.02	0.03	-0.20	0.26
[M_t0_]	Cap Rate qoq	[%]	0.00	0.00	0.00	0.00	0.00
	Vacancy qoq	[%]	0.00	0.00	0.01	-0.03	0.03
	NOI Growth qoq	[%]	0.03	0.04	0.05	-0.33	0.18
	Sold Properties	[#]	617.46	665.00	178.90	182.00	907.00
[M_t-1_]	Cap Rate qoq (lag)	[%]	0.00	0.00	0.00	0.00	0.00
	Vacancy qoq (lag)	[%]	0.00	0.00	0.01	-0.03	0.03
	NOI Growth qoq (lag)	[%]	0.03	0.04	0.05	-0.33	0.18
	Sold Properties (lag)	[#]	610.17	662.00	181.68	182.00	907.00

This table presents the summary statistics of numerical features

**Table 3:** Descriptive Statistics of Categorical Variables

		All Types (N = 7,133)	
	Variable	n	Percent
[P]	Property Type		
	… Apartment	1,904	26.69%
	… Industrial	2,337	32.76%
	… Office	2,056	28.82%
	… Retail	836	11.72%
	Property Subtype		
	… Garden	1,295	18.16%
	… High-rise	455	6.38%
	… Low-rise	154	2.16%
	… Research and Development	120	1.68%
	… Flex Space	412	5.78%
	… Manufacturing	21	0.29%
	… Other	40	0.56%
	… Office Showroom	11	0.15%
	… Warehouse	1,733	24.30%
	… Central Business District	450	6.31%
	… Suburban	1,606	22.52%
	… Community Center	265	3.72%
	… Theme/Festival Center	1	0.01%
	… Fashion/Specialty Center	30	0.42%
	… Neighborhood Center	363	5.09%
	… Outlet Center	2	0.03%
	… Power Center	74	1.04%
	… Regional Mall	34	0.48%
	… Super-Regional Mall	22	0.31%
	… Single-Tenant	45	0.63%
[S]	Appraisal		
	… External	2,485	34.84%
	… Internal	3,079	43.17%
	… Other	1,569	21.99%
	Fund Type		
	… Closed-end Fund	1,370	19.21%
	… ODCE Fund	1,699	23.82%
	… Other	57	0.80%
	… Open-end Fund	1,060	14.86%
	… Single Client Account	2,947	41.32%

This table presents the summary statistics of categorical features

### Models

Non-parametric machine learning methods can identify interactions between the covariates without the need to specify them *a-priori*. Hence, these methods are not limited to any implicit assumptions of the relationship between *X* and *Y* and should be free of manual bias and specification error. To assess whether such methods can add to the understanding of pricing processes beyond the understanding achieved with traditional methods, we attempt to explain the information content in the appraisal errors *Y* using the extreme gradient boosting algorithm (i.e., boosting) by Chen and Guestrin (2016), which is an ensemble of regression trees.

The general concept of a regression tree as introduced by Breiman et al. (1984) is to divide the feature space into mutually exclusive intervals by creating binary decision rules for each feature that contributes to a reduction in the variation of the dependent variable. Such a decision rule is referred to as a split or node and can be thought of as a junction in the process of growing a branch of the tree. This splitting process is continued until the prediction error is minimized or a stopping criterion comes into effect. The resulting leaves of each branch are subsequently referred to as the terminal nodes of the regression tree, each representing a constant value as the final prediction rule. The entirety of these rules can be thought of as the regression tree model. To optimize model performance (i.e., select the optimal hyperparameters for model regularization), a tree model is iteratively trained (i.e., grown) using a training subsample and tested by passing the observations from the respective test subsample down the branches of the tree following the decision rules. Each observation is eventually assigned a terminal leaf corresponding to the final property price prediction.

However, individual trees' intuitiveness and flexibility are accompanied by the risk of quickly overfitting the training sample, thus imposing limitations on unseen data. A more dependable and robust approach is based on the idea of using many individual trees as building blocks of a larger prediction model, known as ensemble learner. The gradient boosting algorithm developed by Friedman (2001) is a prominent example of such ensemble learners. As demonstrated repeatedly in the literature, boosting achieves high accuracy and at the same time consistency for the prediction of property prices in the residential sector, while being comparatively efficient from a computational perspective^12^ (e.g., Mayer et al., 2019; Deppner & Cajias, 2022; Lorenz et al., 2022).

In a boosting algorithm, a single regression tree is fitted as the base model and is then iteratively updated by sequentially growing new regression trees on the residuals of the preceding tree to continue learning and thereby “boosting” model accuracy. The final boosting model consists of an additive expansion of regression trees. The extreme gradient boosting algorithm by Chen and Guestrin (2016) only considers a randomly selected subset from all available predictors at each split in the tree-growing process and is thus a more regularized alternative of the gradient boosting algorithm by Friedman (2001). This introduces an additional source of variation into the model to provide more generalizable and robust estimations.

To further ensure the generalizability of the results, the performance of our models is evaluated using *k*-fold cross-validation. Cross-validation is a resampling technique used to counteract overfitting by partitioning the dataset into *k* mutually exclusive folds of the same size. The model is trained *k* times on *k-1* folds and tested on the* k*^*th*^ fold, respectively*, *such that the model performance is entirely evaluated on unseen data without losing any observations.

By taking the appraisal error as our dependent variable, the manual appraisals from the NPI can be thought of as the base model in our boosting algorithm. Following Pace and Hayunga (2020), we use the standard deviation to measure the total variation in our dependent variable, that is, the manual appraisal error as specified in Eq. (3), as ${\sigma }_{Appraisal}$ and the unexplained residual variation of our boosting estimator as ${\sigma }_{Boosting}$, shown in Eqs. (7) through (9).

7 $${\sigma }_{Appraisal }= \sqrt{\frac{\sum_{i=1}^{n}{\left|y-\overline{y }\right|}^{2}}{n}}$$

8 $${\sigma }_{Boosting }= \sqrt{\frac{\sum_{i=1}^{n}{\left|\varepsilon -\overline{\varepsilon }\right|}^{2}}{n}}$$

9 $$\varepsilon =y- \widehat{y}$$

Our null hypothesis can thus be stated as:

“*The difference between manual appraisals and sales prices cannot be explained by the existing covariates.*”

This is the case when the condition in Eq. (10) is fulfilled.

10 $$H_0:\frac{\sigma_{Appraisal}}{\sigma_{Boosting}}\leq1$$

In other words, this means that deviations between appraisals and sales prices follow a random process, and the improvement provided by machine learning algorithms over existing valuation approaches is not significantly different from zero. In contrast, the alternative hypothesis implies there is structured information content in the deviations between appraisals and sales prices, which machine learning models can exploit to explain these residuals further. This would provide an improvement in the understanding of pricing processes that goes beyond the understanding achieved with current appraisal methods:

- ${\boldsymbol H}_{\mathbf1}\boldsymbol:$ “The difference between manual appraisals and sales prices can be explained by the existing covariates.”

Following the rationale of Pace and Hayunga (2020), the ${H}_{0}$ is rejected when the ratio of the total variation to the residual variation exceeds the value of 1, satisfying the condition in Eq. (11).

11 $${H}_{1}:\frac{{\sigma }_{Appraisal }}{{\sigma }_{Boosting }}>1$$

Considering the results of the ANOVA test, which indicates systematic differences in appraisal errors across property types, we estimate separate models for each of the four asset sectors. Additionally, we calculate one global model for all property types, including the property type as an additional explanatory variable. In total, this results in five models.

After testing our hypotheses, we apply model-agnostic permutation feature importance (Fisher et al., 2019) to all models where the null hypothesis is rejected to examine the structure in appraisal errors. This method yields insights into the decision tree building process of the models so that the features are ranked according to their relative influence in reducing the variation between sales prices and market values and, thus, their contribution to shrinking the appraisal error.

## Empirical Results

This section features the empirical results of our analyses. First, we present the descriptive statistics of the deviation between sales prices and appraisal values of commercial real estate from the NPI. We then examine the variation in these appraisal errors using extreme gradient boosting trees. With respect to our research objectives, we analyze whether appraisal errors contain structured information that tree-based ensemble learners can exploit to further reduce appraisal errors. Subsequently, we discuss the features’ relative importance to infer where the shrinkage in appraisal errors originates.

### Descriptive Statistics

Following Cannon and Cole (2011), we investigate the accuracy and bias in appraisal values as estimates of sales prices. Table 4 provides a summary of the absolute percentage appraisal errors in our sample population and a disaggregated overview for each year and property type. Overall, the MAPE in our sample is 11.1% across all property types and years. This is smaller than the 13.2% reported by Cannon and Cole (2011) for the period between 1984 and 2009 but roughly the same magnitude. On average, accuracy is highest for apartments with an error of 8.6% and lowest for industrial sites with an error of 12.5%. The t-statistic tests the null hypothesis that the MAPE is not significantly different from zero in the respective groupings. The null can be rejected across all years, property types and for the aggregated sample, indicating inaccurate appraisals. We also do not find any evidence that the MAPE has significantly narrowed over the past decade compared to previous years when disregarding the large deviations that occurred during the great financial crisis in 2009.

**Table 4:** Absolute Percentage Error between Sales Price and Manual Appraisal Value

	All Types (N = 7,133)				Apartment (N = 1,904)				Industrial (N = 2,337)				Office (N = 2,056)				Retail (N = 836)			
Year	MdAPE	MAPE	t-Stat		MdAPE	MAPE	t-Stat		MdAPE	MAPE	t-Stat		MdAPE	MAPE	t-Stat		MdAPE	MAPE	t-Stat	
1997	8.02%	9.38%	11.56	***	6.14%	7.04%	5.36	***	8.10%	10.38%	7.78	***	13.25%	13.44%	6.80	***	5.21%	6.87%	4.54	***
1998	11.15%	13.87%	8.96	***	14.14%	11.46%	5.95	***	9.89%	15.21%	3.58	***	15.27%	14.86%	7.94	***	8.78%	11.42%	5.34	***
1999	9.01%	10.12%	14.45	***	6.43%	7.39%	6.65	***	13.54%	11.60%	7.57	***	8.13%	9.71%	7.40	***	13.05%	11.44%	8.20	***
2000	7.65%	9.95%	16.99	***	9.87%	10.63%	11.17	***	5.25%	8.51%	6.87	***	9.13%	10.57%	10.11	***	6.51%	9.60%	5.97	***
2001	6.52%	9.69%	12.29	***	6.70%	8.54%	10.10	***	7.56%	9.91%	6.91	***	6.30%	10.07%	5.35	***	5.02%	10.88%	4.19	***
2002	7.63%	10.87%	12.64	***	7.12%	10.19%	5.62	***	9.30%	12.65%	8.28	***	7.29%	10.18%	6.02	***	8.42%	9.08%	5.98	***
2003	7.14%	9.17%	19.29	***	6.27%	7.94%	9.23	***	6.90%	8.58%	11.31	***	6.48%	9.99%	9.66	***	9.75%	10.83%	10.62	***
2004	8.98%	11.49%	20.21	***	7.85%	9.66%	11.14	***	9.67%	13.28%	10.54	***	8.77%	10.95%	13.08	***	9.59%	11.05%	8.72	***
2005	15.74%	15.97%	29.25	***	9.71%	13.09%	13.90	***	20.20%	17.21%	23.10	***	13.92%	16.32%	10.93	***	17.63%	17.01%	21.11	***
2006	11.43%	13.11%	23.63	***	10.54%	12.36%	12.53	***	12.99%	13.88%	11.77	***	10.78%	13.58%	14.89	***	10.53%	10.52%	7.97	***
2007	10.57%	12.28%	26.97	***	9.28%	11.22%	13.35	***	11.12%	12.14%	18.48	***	11.87%	14.09%	14.78	***	5.21%	8.25%	6.47	***
2008	7.26%	11.96%	8.34	***	7.44%	10.75%	8.29	***	5.91%	7.86%	8.10	***	8.75%	17.75%	4.63	***	5.16%	6.22%	3.49	**
2009	17.32%	22.77%	14.13	***	13.43%	17.51%	9.27	***	20.34%	26.12%	8.10	***	19.19%	27.61%	7.70	***	9.36%	14.49%	3.67	***
2010	9.20%	11.62%	16.70	***	7.87%	10.30%	10.46	***	10.64%	12.90%	9.28	***	9.10%	11.30%	7.64	***	12.62%	12.99%	5.44	***
2011	7.91%	10.53%	18.79	***	6.86%	8.04%	11.84	***	8.98%	10.24%	12.49	***	7.69%	11.32%	8.44	***	8.96%	13.80%	7.49	***
2012	7.02%	10.63%	16.50	***	6.28%	7.74%	11.81	***	7.57%	12.05%	10.16	***	8.08%	12.61%	7.86	***	7.39%	8.05%	7.63	***
2013	7.10%	9.52%	22.82	***	4.08%	5.57%	15.08	***	8.32%	10.90%	15.33	***	9.23%	11.38%	11.25	***	9.38%	11.59%	8.31	***
2014	7.70%	10.57%	19.32	***	5.66%	6.96%	14.28	***	12.62%	12.94%	17.39	***	5.22%	9.69%	7.50	***	5.65%	11.66%	5.12	***
2015	8.47%	11.51%	19.61	***	7.46%	8.59%	15.59	***	10.77%	15.43%	11.29	***	6.64%	10.74%	9.30	***	8.68%	10.45%	8.78	***
2016	6.72%	10.74%	18.53	***	4.78%	7.13%	14.83	***	8.04%	12.43%	14.09	***	6.11%	13.03%	7.97	***	8.12%	9.71%	10.10	***
2017	5.62%	9.09%	15.64	***	4.25%	6.28%	13.99	***	7.50%	11.24%	10.37	***	5.59%	8.37%	11.85	***	4.79%	13.12%	3.00	***
2018	5.96%	8.46%	18.46	***	5.01%	7.21%	12.13	***	7.84%	11.61%	8.57	***	5.73%	7.86%	11.83	***	5.08%	9.09%	3.65	***
2019	6.08%	8.66%	21.56	***	5.57%	6.04%	13.81	***	8.47%	10.61%	16.34	***	5.59%	7.16%	10.23	***	5.29%	9.70%	5.43	***
2020	6.30%	9.29%	14.28	***	3.43%	5.26%	8.79	***	9.75%	10.35%	10.85	***	6.43%	10.66%	6.97	***	7.17%	12.35%	5.80	***
2021	10.32%	13.56%	8.19	***	6.71%	8.45%	4.83	***	14.92%	15.13%	9.43	***	8.40%	14.27%	2.31	**	10.32%	7.26%	2.24	**
All	7.99%	11.12%	81.72	***	6.47%	8.62%	49.70	***	9.73%	12.50%	51.31	***	7.69%	11.71%	39.01	***	8.71%	11.52%	28.86	***

This table presents the median absolute percentage appraisal error (MdAPE) and the mean absolute percentage appraisal error (MAPE) as a measure of accuracy. The t-statistic tests the null hypothesis that the MAPE is not significantly different from zero, i.e., appraisals are accurate

Significance codes indicate that the MAPE is statistically different from zero at the respective level of confidence:

*p* < 0.01 '***', *p* < 0.05 '**', *p* < 0.1 '*'

Subsequently, we examine the signed percentage errors as a metric for bias, which is presented in Table 5. Matysiak and Wang (1995) and Cannon and Cole (2011) state that, on average, positive and negative deviations should cancel out, so appraisals are considered unbiased if the null hypothesis of the t-statistic, that is, the MPE is not significantly different from zero, is accepted. We find this to be the case for some individual years, particularly during flat market phases such as in 2001 and 2002 after the burst of the Dot-com bubble, in 2012 in the aftermath of the great financial crisis, between 2016 and 2017 when capital appreciation in U.S. commercial real estate markets was cooling off, and from 2020 through 2021, when the Covid-19 pandemic caused uncertainty in commercial markets, dampening growth. However, the null hypothesis is rejected for all years in which markets were either in rising or falling regimes. We find that the MPE averages 4.97% during rising markets, indicating a structural underestimation of property prices, whereas this metric turns negative at 12.95% during the sharp downturn between 2008 and 2009, the only period of falling markets in our sample, indicating overestimation of prices. This provides evidence that appraisal values tend to lag sales prices in moving markets and strongly corroborates the findings by Cannon and Cole (2011) and previous studies showing that market cycles have an impact on the reliability of real estate appraisals.

**Table 5:** Signed Percentage Error between Sales Price and Manual Appraisal Value

	All Types (N = 7,133)				Apartment (N = 1,904)				Industrial (N = 2,337)				Office (N = 2,056)				Retail (N = 836)			
Year	MdPE	MPE	t-Stat		MdPE	MPE	t-Stat		MdPE	MPE	t-Stat		MdPE	MPE	t-Stat		MdPE	MPE	t-Stat	
1997	7.22%	6.80%	6.00	***	6.14%	5.97%	3.70	***	7.87%	7.26%	3.82	***	12.36%	10.50%	2.94	**	4.87%	3.73%	1.57	
1998	8.71%	7.60%	3.79	***	14.14%	10.27%	4.17	***	8.46%	3.15%	0.61		15.27%	12.79%	5.50	***	4.75%	2.46%	0.67	
1999	5.46%	5.37%	4.74	***	5.80%	4.62%	2.59	**	6.83%	4.22%	1.39		4.74%	4.83%	2.39	**	10.83%	7.25%	3.25	***
2000	2.69%	2.04%	2.26	**	5.77%	5.74%	3.63	***	1.09%	-1.58%	-0.92		2.09%	1.61%	1.00		4.81%	2.81%	1.16	
2001	2.79%	0.47%	0.43		6.00%	7.04%	6.53	***	3.47%	-0.49%	-0.24		-2.97%	-4.49%	-1.91	*	2.14%	-2.73%	-0.84	
2002	3.21%	0.93%	0.79		4.69%	3.91%	1.73	*	1.23%	-1.89%	-0.86		0.79%	0.25%	0.11		4.56%	3.68%	1.60	
2003	4.24%	3.18%	4.40	***	4.75%	4.10%	3.32	***	3.46%	2.56%	2.13	**	1.31%	1.30%	0.85		8.11%	7.51%	4.38	***
2004	5.23%	4.66%	5.77	***	3.94%	4.58%	3.47	***	3.90%	2.94%	1.69	*	5.83%	5.73%	4.65	***	6.18%	6.99%	3.72	***
2005	14.76%	12.19%	16.84	***	8.95%	10.75%	9.08	***	19.44%	14.05%	12.36	***	10.93%	8.56%	4.45	***	17.06%	16.19%	16.76	***
2006	9.80%	9.26%	11.98	***	9.79%	10.89%	9.09	***	9.96%	6.65%	3.73	***	9.86%	10.62%	8.79	***	8.68%	6.80%	3.19	***
2007	8.47%	7.85%	11.82	***	7.42%	6.82%	5.41	***	9.28%	8.44%	8.52	***	8.67%	8.28%	5.90	***	4.55%	6.36%	3.88	***
2008	-1.85%	-5.44%	-3.26	***	-3.94%	-2.33%	-1.12		-2.99%	-4.89%	-3.80	***	0.85%	-8.87%	-2.02	**	-2.68%	-1.99%	-0.62	
2009	-16.87%	-20.46%	-11.40	***	-13.20%	-14.94%	-6.64	***	-20.34%	-24.66%	-7.19	***	-19.19%	-24.47%	-5.93	***	-7.67%	-12.41%	-2.61	**
2010	4.50%	2.55%	2.34	**	6.24%	7.01%	5.16	***	-1.35%	-1.14%	-0.51		5.93%	2.20%	0.95		3.08%	-1.11%	-0.29	
2011	4.28%	3.21%	3.80	***	5.72%	4.09%	3.77	***	4.61%	3.14%	2.35	**	3.75%	3.56%	1.74	*	-1.05%	1.73%	0.63	
2012	1.41%	-0.68%	-0.82		2.21%	1.06%	1.08		2.02%	-0.29%	-0.19		-1.38%	-3.34%	-1.65	*	-2.13%	-0.45%	-0.27	
2013	3.42%	2.96%	5.09	***	2.69%	3.26%	6.23	***	4.86%	4.07%	3.80	***	3.01%	1.74%	1.21		3.53%	1.90%	0.96	
2014	5.28%	4.45%	6.41	***	4.50%	4.11%	5.69	***	10.75%	6.97%	6.44	***	2.19%	1.83%	1.20		3.78%	2.91%	1.07	
2015	4.57%	2.89%	3.65	***	6.72%	6.36%	8.48	***	4.75%	-0.31%	-0.16		3.17%	1.60%	1.07		5.29%	5.81%	3.36	***
2016	0.75%	-1.14%	-1.53		3.35%	3.99%	5.89	***	-3.19%	-3.47%	-2.69	***	-1.65%	-5.06%	-2.64	***	2.36%	2.00%	1.20	
2017	1.83%	0.38%	0.52		3.35%	3.59%	5.59	***	1.78%	-0.23%	-0.16		-0.84%	-0.10%	-0.10		-0.65%	-6.74%	-1.42	
2018	2.76%	1.87%	2.93	***	3.94%	3.70%	4.49	***	4.78%	2.70%	1.41		1.25%	0.57%	0.61		-2.58%	-4.43%	-1.36	
2019	2.58%	2.62%	4.62	***	1.11%	0.59%	0.76		6.10%	6.95%	7.87	***	1.60%	1.33%	1.37		-3.74%	-7.37%	-3.66	***
2020	0.53%	-0.39%	-0.42		1.36%	1.71%	1.93	*	4.08%	4.89%	3.16	***	-2.90%	-3.26%	-1.61		-5.36%	-8.17%	-3.04	***
2021	3.21%	0.87%	0.37		6.42%	4.67%	1.59		5.49%	3.29%	1.13		-1.65%	-6.64%	-0.93		-10.32%	-7.26%	-2.24	
All	3.78%	2.71%	14.51	***	4.09%	4.07%	16.57	***	4.60%	2.62%	7.46	***	2.53%	1.47%	3.72	***	3.76%	2.90%	5.23	***

This table presents the median percentage appraisal error (MdPE) and the mean percentage appraisal error (MPE) as a measure of biasedness. The t-statistic tests the null hypothesis that the MPE is not significantly different from zero, i.e., appraisals are unbiased

Significance codes indicate that the MPE is statistically different from zero at the respective level of confidence:

*p* < 0.01 '***', *p* < 0.05 '**', *p* < 0.1 '*'

### Residual Standard Deviation

After confirming the findings of inaccuracy and structural bias made by Cannon and Cole (2011) for our sample period, we investigate the variation in the respective appraisal errors (i.e., residuals). The results of the analysis were obtained by applying the extreme gradient boosting algorithm (i.e., boosting) separately for each property type and to the aggregated dataset. The models were repeatedly cross-validated by ten mutually exclusive folds to avoid overfitting, such that each of the folds was used once as a test sample. The hyperparameters of the boosting estimators were optimized via the root mean square error using a grid search procedure. All error measures are reported as 10-fold cross-validation errors, thus representing out-of-sample estimations. The results are displayed in Table 6. By analogy to the study of Pace and Hayunga (2020), the last two columns depict the ratio of the standard deviation from the dependent variable (i.e., total variation of appraisal errors) to the residuals resulting from the machine learning estimations (i.e., unexplained variation of appraisal errors). The ratio exceeds 1 for any case where the appraisal errors can be further explained by the applied boosting procedure.

**Table 6:** Residual Standard Deviation

	${\upsigma }_{Appraisal}$	${\upsigma }_{Boosting}$	${{\mathrm{R}}^{2}}_{Boosting}$	$\frac{{\upsigma }_{Appraisal }}{{\upsigma }_{Boosting}}$
All Types	0.15	0.13	0.26	1.17
Apartment	0.11	0.09	0.31	1.20
Industrial	0.16	0.14	0.28	1.18
Office	0.16	0.14	0.25	1.16
Retail	0.15	0.13	0.22	1.14

This table benchmarks the residual variation of manual appraisals against the residual variation of the boosting algorithm, whereby σ is the standard deviation of the respective residuals. A performance improvement occurs whenever the ratio of ${\upsigma }_{Appraisal}$ over ${\upsigma }_{Boosting}$ exceeds the value 1

**Fig. 2:**
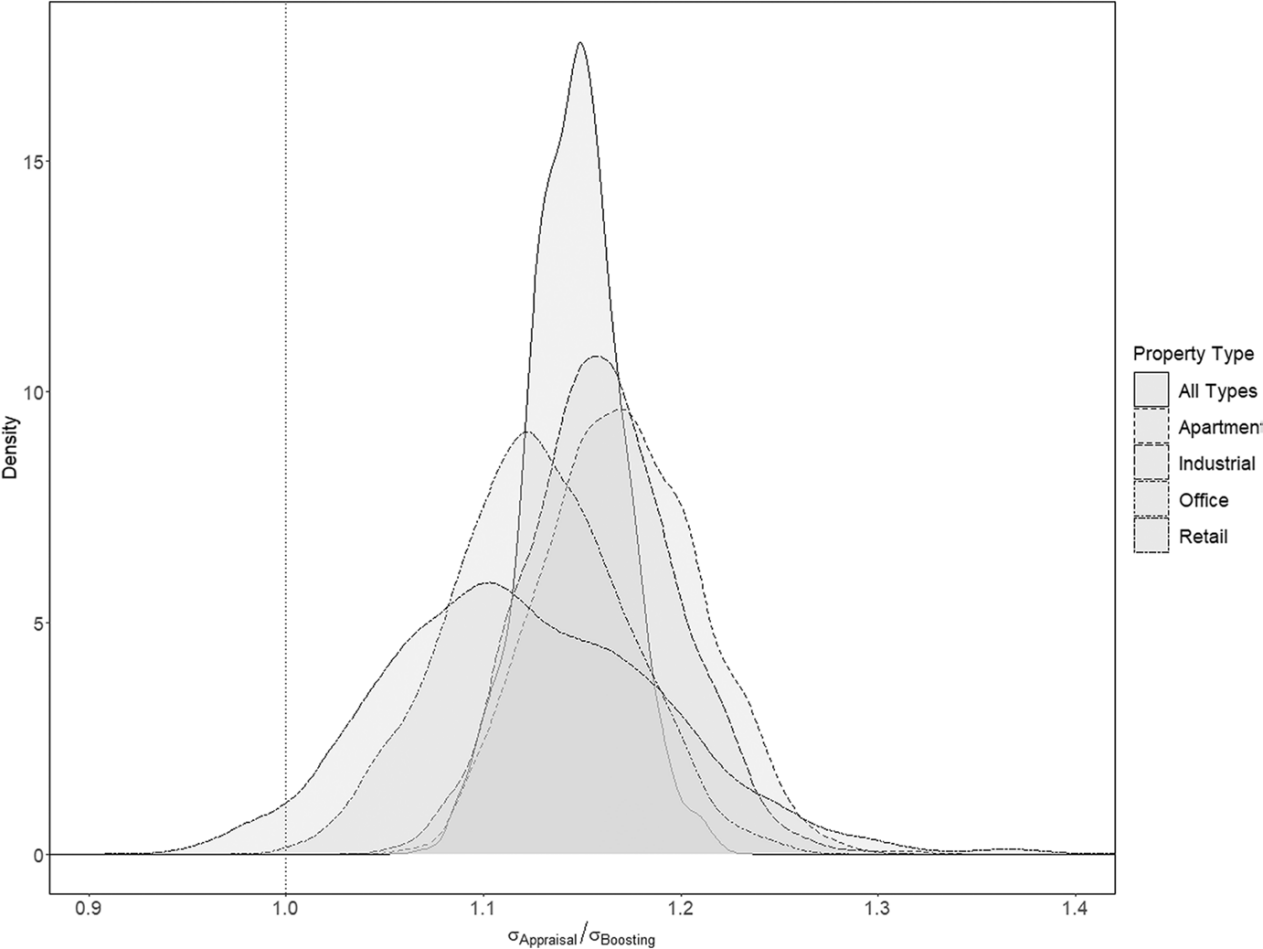
Bootstrap Distribution of Model Performance. Notes: The density plot shows the bootstrap distribution of the model performance for all five models using 1,000 random bootstrap samples. A performance improvement occurs whenever the ratio $\frac{{\upsigma }_{\mathrm{Appraisal }}}{{\upsigma }_{\mathrm{Boosting }}} >1$, as indicated by the dotted horizontal line. The area to the right of the dotted line can be interpreted as the confidence interval for which the null hypothesis $\frac{{\upsigma }_{\mathrm{Appraisal }}}{{\upsigma }_{\mathrm{Boosting }}} \le 1$ can be rejected. The null hypothesis can be rejected at a 5% level of significance for all models and at a 1% level of significance for all models except for the retail model. The respective ratios measured by 10-fold cross-validation are presented in Table 6

We find the results in Table 6 to be unequivocal in all four asset classes, as a reduction in the variation of appraisal errors (i.e., residual variation) can be achieved in all cases. The boosting algorithms yield considerable improvements, with coefficients taking values well above 1.^13^ The reduction in the residual variation is highest for apartments with 20.5% and lowest for retail properties with approximately 14.2%. By implication, such a reduction signals that the appraisal error is systematic to some extent rather than purely random. To formally test our hypothesis and rule out that improvements occur by pure chance, we apply bootstrapping to create confidence intervals for the shrinkage of the residual variation in our dependent variable. This is achieved by generating 1,000 random bootstrap samples and repeatedly training and testing the models on each sample. Figure 2 presents the bootstrap distribution of the model performance for all five models. Based on the bootstrap confidence intervals, the null hypothesis stated in Eq. (10) can be rejected at a 5% level of significance for the retail model and at a 1% level of significance for all other models.

**Fig. 3:**
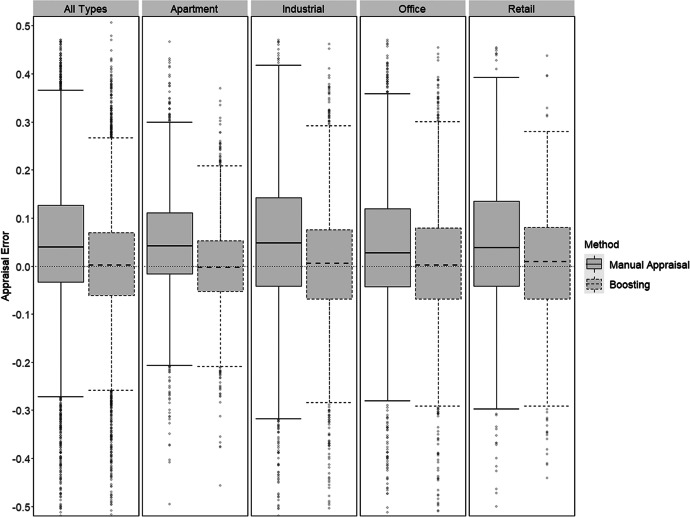
Comparison of Residual Variation. Notes: The boxplots show the distribution of the raw appraisal errors (solid line) in comparison to the boosted appraisal errors (dashed line). The box of each boxplot represents 50% of the data within the 25^th^ and 75^th^ percentile. The bold line within the box indicates the median of each distribution. The whiskers indicate the 1.5 interquartile range (IQR). The dotted horizontal line marks the null point on the y-axis

Figure 3 depicts the distributions of the residuals by asset class. Matysiak and Wang (1995) and Cannon and Cole (2011) show appraisal errors to be biased in their samples. That is, the mean of the error distribution was positive or negative and not around zero. This can also be observed in Fig. 3 for the median appraisal errors, which are considerably above the horizontal null point line in all asset classes, indicating that most properties are overvalued. In contrast, all machine learning models produce residuals close to zero. This indicates that the estimated models are not biased and produce reliable responses. Furthermore, the 25th and 75th percentiles of the boxplots show that the dispersion of the residuals from boosting is smaller than the original appraisal errors for all property types.

We also see a relationship between the homogeneity of asset classes and the performance improvement. Relatively homogenous property types (i.e., apartments, industrial) benefit more from machine learning than relatively heterogenous asset classes (i.e., retail, office). The same applies to the sample size, as data-driven techniques require homogenous and large samples to learn patterns from the data.

To test whether the reduction in the residual variation can also reduce bias in the actual appraisals, we infer hypothetical appraisal values from the estimated percentage appraisal errors by multiplying these by the original appraisal values. In analogy to the descriptive statistics of the manual appraisal errors in the "Appraisal Error" section, Tables 7 and 8 present the adjusted appraisal values obtained by the boosting algorithms. Overall, the MAPE presented in Table 7 is reduced for all asset classes. In the aggregated models, a reduction from 11.12% to 9.25% is achieved. The highest absolute reduction in the MAPE was achieved for industrial properties with 2.48 percentage points (i.e., 19.85%) by the boosting model. The highest relative reduction in the MAPE was achieved for apartments with 20.91% (i.e., 1.80 percentage points). The lowest absolute and relative improvement can be observed for office buildings. However, this is still 1.44 percentage points absolute and above 12.32% relative. These figures confirm the findings of a significant reduction in the residual variation (see Table 6) and support the hypothesis that machine learning algorithms can exploit the structured covariance found in the residuals to further shrink appraisal errors.

**Table 7:** Absolute Percentage Error between Sales Price and Boosting Appraisal Value

	All Types (N = 7,133)				Apartment (N = 1,904)				Industrial (N = 2,337)				Office (N = 2,056)				Retail (N = 836)			
Year	MdAPE	MAPE	t-Stat		MdAPE	MAPE	t-Stat		MdAPE	MAPE	t-Stat		MdAPE	MAPE	t-Stat		MdAPE	MAPE	t-Stat	
1997	5.88%	7.63%	9.07	***	5.37%	5.76%	5.67	***	7.06%	8.03%	5.79	***	8.83%	11.03%	4.07	***	6.41%	5.71%	4.32	***
1998	8.54%	11.24%	6.94	***	6.50%	7.09%	5.97	***	9.05%	14.30%	2.99	***	9.17%	10.46%	8.99	***	7.51%	9.53%	4.72	***
1999	6.86%	8.55%	13.47	***	5.17%	6.41%	4.81	***	10.87%	11.88%	8.36	***	7.24%	8.25%	7.75	***	6.86%	7.94%	6.46	***
2000	6.43%	8.69%	15.34	***	6.51%	7.43%	8.97	***	5.60%	8.49%	6.67	***	8.47%	10.45%	9.54	***	7.94%	10.13%	5.75	***
2001	5.77%	8.62%	11.86	***	5.05%	6.22%	8.60	***	7.00%	9.30%	6.81	***	5.70%	8.77%	5.21	***	5.83%	11.90%	4.62	***
2002	7.13%	10.29%	11.34	***	6.69%	8.89%	4.41	***	9.00%	12.36%	7.89	***	5.94%	8.67%	5.97	***	5.94%	8.47%	6.56	***
2003	6.07%	8.24%	17.01	***	4.80%	6.93%	7.25	***	5.98%	7.78%	8.72	***	7.23%	9.10%	9.59	***	7.52%	8.04%	7.68	***
2004	7.64%	10.39%	18.16	***	7.62%	7.95%	11.45	***	8.78%	12.60%	9.57	***	8.25%	9.47%	13.28	***	9.10%	9.87%	10.90	***
2005	8.83%	11.33%	19.01	***	7.58%	9.51%	11.46	***	7.82%	10.67%	13.30	***	9.25%	12.64%	10.87	***	8.41%	9.41%	13.14	***
2006	8.06%	10.20%	21.02	***	7.17%	8.59%	11.40	***	8.01%	10.00%	11.49	***	9.32%	11.10%	13.34	***	7.75%	8.96%	6.87	***
2007	6.93%	9.03%	21.85	***	6.49%	8.44%	11.88	***	7.28%	8.47%	13.41	***	8.66%	11.30%	11.85	***	7.18%	8.04%	7.75	***
2008	6.19%	10.00%	8.14	***	8.16%	9.05%	7.78	***	4.15%	6.27%	7.82	***	9.33%	16.24%	4.80	***	8.83%	8.07%	5.00	***
2009	10.16%	12.82%	13.56	***	8.57%	9.98%	10.04	***	10.45%	14.62%	7.19	***	9.53%	13.01%	6.41	***	4.97%	11.64%	3.24	***
2010	8.21%	10.12%	15.29	***	7.30%	8.85%	9.69	***	9.02%	10.46%	8.52	***	8.01%	10.04%	7.87	***	10.28%	13.18%	6.37	***
2011	6.65%	9.12%	16.26	***	4.27%	6.77%	9.10	***	6.11%	8.00%	9.88	***	8.75%	11.35%	8.29	***	6.75%	11.62%	6.71	***
2012	6.32%	9.36%	17.82	***	5.35%	6.75%	11.30	***	6.10%	10.28%	10.13	***	7.76%	10.80%	9.77	***	6.94%	7.64%	7.96	***
2013	6.18%	8.89%	21.72	***	2.82%	4.87%	12.25	***	7.68%	9.43%	14.58	***	8.10%	10.79%	11.25	***	8.27%	12.16%	7.31	***
2014	5.88%	8.69%	16.04	***	5.52%	6.31%	14.90	***	5.77%	9.16%	12.56	***	6.33%	9.55%	7.51	***	6.92%	11.16%	4.76	***
2015	6.76%	9.26%	19.35	***	5.28%	6.52%	14.07	***	8.99%	12.23%	10.66	***	6.52%	9.74%	10.15	***	7.50%	8.43%	8.57	***
2016	6.33%	9.21%	20.62	***	4.17%	5.26%	14.38	***	7.98%	10.88%	12.81	***	6.82%	11.74%	9.80	***	7.32%	8.90%	9.29	***
2017	5.58%	8.82%	14.08	***	3.74%	5.18%	13.14	***	8.03%	11.97%	9.39	***	6.25%	8.64%	11.36	***	3.04%	12.04%	2.84	***
2018	5.96%	8.02%	17.42	***	4.45%	5.77%	10.82	***	7.91%	10.80%	7.49	***	6.00%	7.61%	11.98	***	6.87%	10.44%	3.44	***
2019	4.91%	6.92%	20.00	***	4.78%	5.63%	11.87	***	4.38%	7.22%	12.50	***	5.54%	7.09%	11.22	***	5.24%	9.48%	6.02	***
2020	5.40%	7.90%	14.15	***	4.36%	5.42%	8.83	***	5.19%	7.22%	8.55	***	8.28%	10.76%	7.89	***	7.72%	10.80%	6.58	***
2021	10.40%	12.27%	8.39	***	3.51%	7.15%	2.97	**	13.39%	13.26%	10.17	***	9.30%	16.09%	2.86	***	7.26%	5.34%	2.25	
All	6.58%	9.25%	75.61	***	5.24%	6.82%	46.47	***	7.27%	10.02%	44.60	***	7.49%	10.27%	41.11	***	7.48%	9.89%	25.83	***

This table presents the boosting-adjusted median absolute percentage appraisal error (MdAPE) and the mean absolute percentage appraisal error (MAPE) as a measure of accuracy. The t-statistic tests the null hypothesis that the MAPE is not significantly different from zero, i.e., appraisals are accurate

Significance codes indicate that the MAPE is statistically different from zero at the respective level of confidence:

*p* < 0.01 '***', *p* < 0.05 '**', *p* < 0.1 '*'

**Table 8:** Signed Percentage Error between Sales Price and Boosting Appraisal Value

	All Types (N = 7,133)				Apartment (N = 1,904)				Industrial (N = 2,337)				Office (N = 2,056)				Retail (N = 836)			
Year	MdPE	MAPE	t-Stat		MdPE	MAPE	t-Stat		MdPE	MAPE	t-Stat		MdPE	MAPE	t-Stat		MdPE	MAPE	t-Stat	
1997	0.29%	0.31%	0.25		-1.34%	-0.17%	-0.10		0.66%	1.14%	0.57		5.73%	3.70%	0.81		0.21%	0.54%	0.24	
1998	3.00%	-0.18%	-0.09		1.51%	1.23%	0.51		4.48%	-1.33%	-0.24		4.07%	2.97%	1.37		2.47%	-1.95%	-0.61	
1999	-1.07%	-0.58%	-0.53		-1.38%	-2.13%	-1.08		4.12%	-0.36%	-0.11		0.49%	0.15%	0.08		0.35%	-0.82%	-0.42	
2000	-0.35%	-1.18%	-1.42		-2.17%	-1.47%	-1.11		-1.04%	-2.50%	-1.44		-0.72%	-1.80%	-1.11		0.06%	-1.58%	-0.60	
2001	0.66%	-1.35%	-1.39		0.49%	0.24%	0.22		3.28%	-1.88%	-1.00		-1.93%	-3.03%	-1.43		1.47%	-3.11%	-0.92	
2002	0.29%	-1.20%	-1.02		-1.26%	-2.39%	-1.01		1.03%	-1.63%	-0.74		-0.38%	-0.78%	-0.41		1.39%	1.30%	0.60	
2003	0.47%	-0.21%	-0.30		-0.03%	-0.60%	-0.46		-0.86%	-0.95%	-0.76		-1.09%	-0.88%	-0.63		1.16%	-0.12%	-0.07	
2004	0.05%	-0.99%	-1.23		-1.22%	-0.78%	-0.68		-0.92%	-2.70%	-1.55		0.39%	-0.62%	-0.54		2.70%	0.69%	0.38	
2005	1.22%	-1.00%	-1.27		-1.20%	-0.35%	-0.28		3.40%	0.58%	0.48		1.52%	-0.72%	-0.45		1.87%	-0.13%	-0.11	
2006	0.25%	-0.44%	-0.57		0.44%	0.36%	0.28		1.43%	0.55%	0.39		-0.22%	0.20%	0.15		-0.10%	-0.02%	-0.01	
2007	-0.14%	-0.47%	-0.75		0.04%	-0.28%	-0.25		1.36%	-0.09%	-0.10		0.94%	0.19%	0.13		0.10%	1.14%	0.61	
2008	-0.24%	-2.07%	-1.42		-0.81%	-1.50%	-0.82		-1.21%	-1.74%	-1.51		1.85%	-5.00%	-1.25		-2.75%	-2.68%	-0.71	
2009	3.25%	-1.51%	-1.09		-0.98%	-1.10%	-0.67		1.87%	-3.89%	-1.38		0.11%	-3.73%	-1.31		-2.45%	-4.87%	-0.94	
2010	0.71%	0.28%	0.28		2.55%	1.80%	1.28		-1.15%	-0.24%	-0.13		3.16%	2.47%	1.23		6.48%	0.55%	0.15	
2011	0.88%	-0.46%	-0.57		1.34%	-0.81%	-0.73		1.00%	-0.34%	-0.29		-1.41%	-0.86%	-0.40		0.52%	-0.57%	-0.24	
2012	0.22%	-0.89%	-1.28		0.28%	-1.07%	-1.23		-0.64%	-1.91%	-1.48		0.94%	-1.24%	-0.80		-1.47%	-0.13%	-0.08	
2013	0.10%	-0.68%	-1.19		0.48%	-0.46%	-0.82		-0.59%	-0.82%	-0.83		2.01%	-0.74%	-0.54		0.65%	-0.63%	-0.28	
2014	0.33%	-0.81%	-1.22		-0.93%	-0.49%	-0.67		0.99%	-0.51%	-0.52		0.55%	-0.81%	-0.53		0.37%	-2.02%	-0.73	
2015	-0.04%	-0.67%	-1.03		-0.43%	-0.21%	-0.28		-1.44%	-2.59%	-1.68	*	0.38%	-0.82%	-0.63		1.86%	1.86%	1.21	
2016	-0.08%	-1.01%	-1.68	*	-0.24%	0.04%	0.07		-0.84%	-1.43%	-1.19		-0.54%	-3.07%	-2.02	**	1.11%	-0.05%	-0.03	
2017	-0.13%	-1.30%	-1.72	*	-0.34%	-0.25%	-0.40		-0.71%	-2.53%	-1.56		-1.41%	-1.11%	-1.02		1.13%	-6.07%	-1.33	
2018	-0.73%	-1.17%	-1.87	*	-0.36%	-0.97%	-1.30		0.28%	-0.88%	-0.46		-0.71%	-1.35%	-1.50		-1.93%	-5.99%	-1.58	
2019	-0.24%	-0.47%	-0.98		-0.04%	-1.16%	-1.54		0.65%	0.59%	0.75		-0.32%	-0.59%	-0.64		0.71%	-3.39%	-1.65	
2020	-0.05%	-0.44%	-0.56		-1.48%	-0.47%	-0.51		0.51%	0.61%	0.47		-1.48%	-1.35%	-0.69		-2.49%	-3.22%	-1.31	
2021	-0.13%	-0.99%	-0.47		1.96%	1.29%	0.38		-0.43%	-0.39%	-0.15		2.66%	-3.95%	-0.56		-0.63%	-0.50%	-0.11	
All	0.17%	-0.81%	-4.94	***	-0.27%	-0.49%	-2.28	**	0.46%	-1.01%	-3.30	***	0.09%	-1.03%	-3.06	***	0.85%	-1.00%	-1.96	**

This table presents the boosting-adjusted median percentage appraisal error (MdPE) and the mean percentage appraisal error (MPE) as a measure of biasedness. The t-statistic tests the null hypothesis that the MPE is not significantly different from zero, i.e., appraisals are unbiased

Significance codes indicate that the MPE is statistically different from zero at the respective level of confidence:

*p* < 0.01 '***', *p* < 0.05 '**', *p* < 0.1 '*'

Compared to Table 5, the mean percentage errors in Table 8 reveal that the bias in appraisal values could be successfully eliminated in most of the years and asset sectors. The acceptance of the null hypothesis that the MPE is not significantly different from zero for all the years except for the period between 2016 and 2018, in which the null could only be rejected at the 10% confidence level, confirms that manual appraisal errors are systematic. It also further supports previous findings in that the boosting estimator provides unbiased estimates, although the mean percentage errors are negative for all years except for 1997 and 2010, indicating a slight overestimation of the inferred appraisal values.

Overall, we find that boosting can provide material improvements in increasing accuracy and reducing structural bias in commercial appraisal values. However, it should also be mentioned that machine learning methods are no crystal ball that can accurately predict downturns such as during the great financial crisis without previously learning the effects of varying economic conditions under transitioning market regimes. Moreover, external shocks such as pandemics, wars, or any sort of crises are difficult to train since they occur infrequently and can take on various forms.

### Permutation Feature Importance

**Fig. 4:**
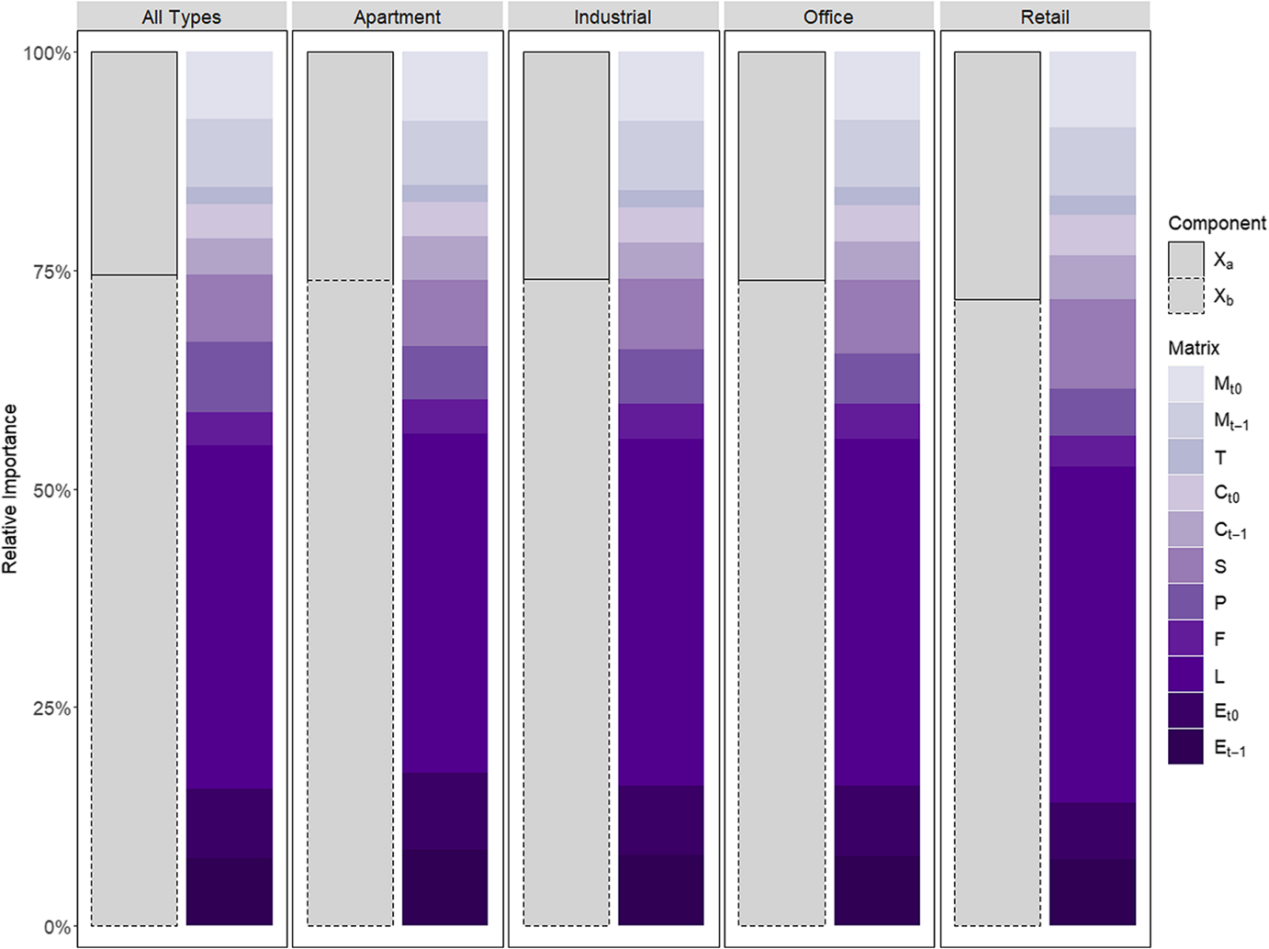
Relative Permutation Feature Importance. Notes: The bar chart shows the relative permutation feature importance of both components X_a_ and X_b_ (indicated by the linetype) and the various feature clusters described in the "Explanatory Variables" section (indicated by the color) for each of the five models. The relative importance on the y-axis indicates the relative contribution of each component and cluster to the reduction of the prediction error. The order of groupings is arbitrary

To draw conclusions about which features contribute most to the shrinkage of the residual variation, we apply the model-agnostic permutation feature importance by Fisher et al. (2019). Figure 4 provides a summary of the feature groupings introduced in the "Explanatory Variables" section, decomposed according to their relative importance in shrinking the appraisal error. Features that repeatedly appear at early splitting points of the individual regression trees or show up more often in the tree-growing process have a high importance score. Identifying these features provides insights into factors that are not adequately reflected in current appraisal practices. This can offer constructive criticism to improve the state-of-the-art (Pace & Hayunga, 2020).

The bar chart in Fig. 4 shows that both components *a* and *b* have an evident influence on appraisal errors, with component *b* dominating by about three-quarters. This indicates that the improvement achieved by the boosting algorithm is not solely due to the time lag between appraisal and sale, but results to a great extent from valuation bias.

Overall, location (*L)* appears to be the most relevant cluster for explaining appraisal errors, accounting for nearly 40% across all models. To a great extent, this is driven by the spatial coordinates. When a regression tree splits on the latitude and longitude, it effectively identifies new submarkets for which it generates individual models, indicating that spatial considerations on the micro-level are not appropriately reflected in appraisal values. This is consistent with Pace and Hayunga (2020), who find that the performance improvement of boosting and bagging regression trees compared to linear hedonic models results to a great extent from exploiting spatial structures in the residuals that cannot be captured with location dummies, such as ZIP code or MSA code areas. However, this seems to be different for industrial properties, as the resolution of MSAs appears to exploit spatial structures in the residuals better than the coordinates, implying that locational factors on the macro-level are overlooked in this sector.

With respect to component *a,* we find Capex in the second quarter before the sale to be the feature with the highest average impact on appraisal errors across all models. This is surprising, as the appraiser should know Capex measures before they occur. However, Beracha et al. (2019) find that in instances, appraisals are updated by simply adding Capex to the market values. This is known as a stale appraisal and may not adequately reflect the true intrinsic value of a building improvement.

For component *b*, the building occupancy is on average the most important feature driving appraisal errors. As described by Beracha et al. (2019), the relation between vacant space and commercial real estate value depends on the optionality of vacant space, which can be based on either a growth hypothesis (i.e., assuming higher future NOI growth from the potential of leasing up vacant space) or a risk hypothesis (i.e., assuming idiosyncratic weaknesses and higher uncertainty in future NOI growth due to vacant space). Differences between valuations and sales prices can occur depending on whether appraisers and investors see vacant space as an upside potential related to rental growth or as a downside potential associated with uncertainty. Consistent with our findings on the systematic overvaluation of appraisals in the "Descriptive Statistics" section, Beracha et al. (2019) demonstrate that, on average, the option value of vacant space is overvalued, which is not surprising as buyers may incorporate more risks than sellers aiming to achieve a higher sale price.

Based on Cannon and Cole (2011), we also control for appraisal type and fund type. The authors expect internal appraisals to be less accurate than external appraisals and properties owned by open-end funds to be more accurate than closed-end funds or separate account properties. This is because internal appraisers tend to be less objective and more likely to smooth appraisals, and open-end funds rely on higher appraisal accuracy as investors can trade in and out based on the appraised values, thus allowing informed investors to gain excess returns if the deviation between appraised values and market values is too high (Cannon & Cole, 2011). The authors confirm that appraisal errors are smaller for properties held in open-end funds than properties owned by closed-end funds and separate accounts. However, they find no evidence that external appraisals from an independent third party are significantly lower than internal appraisals. These findings are consistent to our feature importance, as the fund type has a moderate average influence in explaining appraisal errors, while the appraisal type is, on average, the least important feature across all models, implying no significant impact on the predictions of the models.

## Conclusion

Accurate and timely valuations are important to stakeholders in the real estate sector, including authorities, banks, insurers as well as pension and sovereign wealth funds. They form the basis for informed decisions on financing, developing portfolio strategies and undertaking transactions, as well as for reporting to boards, investors, and tax offices. However, research has shown that, over the past 40 years, commercial real estate appraisals have had a consistent tendency of structural bias and inaccuracy, while lagging true market dynamics (Cole et al., 1986; Webb, 1994; Matysiak & Wang, 1995; Fisher et al., 1999; Cannon & Cole, 2011). While traditional appraisal methods used in the commercial sector have by and large remained the same for decades, statistical learning methods have become increasingly popular. These methods have demonstrated their potential to accurately capture quickly changing market dynamics and complex pricing processes in the residential property sector. However, the transfer of such data-driven valuation methods to commercial real estate faces significant challenges such as data scarcity, heterogeneity, and opaqueness of the models. This poses the question of whether machine learning algorithms can provide material improvement to state-of-the-art appraisal practices in commercial real estate with respect to accuracy and bias of valuations.

Using property-level transaction data from 7,133 properties included in the NCREIF Property Index (NPI) between 1997 and 2021 across the United States, we analyze whether deviations between appraisal values and subsequent transaction prices in the four major commercial real estate sectors (apartment, industrial, office, and retail) contain structured variation that can be further explained by advanced machine learning methods. We find that extreme gradient boosting trees can substantially decrease the variation in appraisal errors across all four property types, thereby increasing accuracy and eliminating structural bias in appraisal values. Improvements are greatest for apartments and industrial properties, followed by office and retail buildings. To clarify where the improvements originate, we employ model-agnostic permutation feature importance and show the features’ relative importance in explaining appraisal errors. We find that especially spatial and structural covariates have a dominant influence on appraisal errors, while only one-fourth of the explained variation can be attributed to the time lag between the appraisal and sale date.

The results of our study indicate that current appraisal practices leave room for improvement, which machine learning methods can exploit to provide additional guidance for commercial real estate valuation. The use of such algorithms can make valuations more efficient and objective while being less susceptible to subjectivity and receptive to a wider range of information. Moreover, these methods offer regulatory bodies and central banks the opportunity to quickly analyze and forecast real estate price developments to detect early signs of price bubbles, stress-test the banking system’s stability in shock scenarios or assess the impact of interest rate decisions and rent controls.

Despite their potential for many areas in the industry, machine learning algorithms also encounter limitations that should be carefully considered before their use, as they are not a panacea for all problems in the sector. While algorithms can reduce bias and increase objectivity, they are still developed and trained by humans and thus, remain subject to bias to some extent. In this context, data availability is currently one of the most critical problems for the use of machine learning in the commercial real estate sector, since the complex architectures of the models require substantial amounts of representative training data to produce unbiased and reliable results. Moreover, it should be mentioned that, although the methods can produce accurate predictions of property values by finding patterns between input and output data, they do not consider the laws of economics and thus, cannot justify the rationale behind these patterns or determine causality in the relation between input and output data. This issue is amplified by the lack of inherent interpretability of these models, as they are opaque black boxes that do not provide inference. Although this can be partly circumvented with model-agnostic interpretation techniques, these methods have their very own limitations and pitfalls, and high computational expense can be another limiting factor for their practical implementation.

That said, algorithms can excel humans in quickly learning relationships from large amounts of data, but they have no economic justification and cannot consider aspects that require reasoning. If applied prudently, these methods can add to an enhanced ex ante understanding of pricing processes that may support valuers in the industry and contribute to more dependable and efficient valuations in the future. Yet, we do not believe that machine learning algorithms can substitute the profession of appraisers any time soon due to the restrictions mentioned above as well as regulatory and ethical challenges.

Having demonstrated the potential of machine learning for many areas of the industry, while at the same time raising awareness for the limitations of these techniques, we hope to stimulate further research that contributes to the development of algorithmic approaches in this field. Such research may, for instance, address the exact relations between features and property prices to offer further guidance for the appraisal industry.

## Data Availability

The data were provided by the National Council of Real Estate Investment Fiduciaries (NCREIF) and are confidential.

## Footnotes

1 Implicit in this definition is the consummation of a sale as of a specified date and the passing of title from seller to buyer under conditions whereby: (1) Buyer and seller are typically motivated; (2) Both parties are well informed or well advised, and acting in what they consider their own best interests; (3) A reasonable time is allowed for exposure in the open market; (4) Payment is made in terms of cash in U.S. dollars or in terms of financial arrangements comparable thereto; and. (5) The price represents the normal consideration for the property sold unaffected by special or creative financing or sales concessions granted by anyone associated with the sale. 12 C.F.R. § 34.42 (2022).

2 The NPI is a quarterly index tracking the performance of core institutional property markets in the U.S.

3 This includes commingled real estate funds (open and closed-end), separate accounts, individual accounts, private REITs, REOCs, and joint-venture partnerships.

4 For further details, refer to the NCREIF PREA Reporting Standards at www.reisus.org.

5 When we calculate the mean absolute percentage errors for the second quarter before sale, we observe market values that deviate from sale prices by up to 377%. We crop the distribution of percentage errors at the 99^th^ percentile, thus allowing for deviations by up to 60%.

6 After data cleaning, we observe sale prices per square foot between $0.8 and $915,501.1 indicating potential data errors. To keep data loss at a minimum, we crop the distributions at the lower 0.5^th^ and the upper 99.5^th^ percentiles.

7 The sample includes 61 observations for which the building age takes values between 101 and 157 years, most of which are unique. We assign those observations the value 100, thus effectively creating a partition for buildings that are older than 100 years, so the trees cannot overfit single observations by using unique building ages.

8 This excludes tenant improvements, lease commissions, and additional acquisition costs, which are incentives or fees that do not affect the quality of a property.

9 The remaining 7% result mainly from missing or incomplete addresses.

10 Of which 1,904 are apartments, 2,337 are industrial, 2,056 are office and 836 are retail.

11 In a similar study by Cannon and Cole (2011), the authors start with 9,439 properties for a period of 25 years and, after filtering, end up with a sample of 7,214 sales. The relative data loss is higher in our case, as we use substantially more covariates with missing entries that result in data leakage.

12 Estimations were executed on a standard 1.80 GHz processor with four cores, eight logical processors and eight gigabytes of RAM using a 64-bit Windows operating system. Hyperparameter tuning for optimization of the boosting models required between 25 and 64 h for each of the four property types, running in parallel. The model including all four property types required 116.5 h of computation time. Hyperparameter tuning was performed via a grid search procedure with 1,000 evaluations and 10-fold cross-validation. The training and testing of the optimized boosting models via 10-fold cross-validation took between 1.5 and 3.8 min for each of the four property types and 7 min for the aggregated model.

13 We have considered and tested a random forest regression (i.e., bagging) next to the extreme gradient boosting algorithm (i.e., boosting) and found no material difference in the explanatory power between the boosting and bagging estimators (${\upsigma }_{Bagging}$ was on par with ${\upsigma }_{Boosting}$ up to the second decimal place for all models and up to the third decimal place for all models except for office with a deviation of 0.001). However, computation time for bagging was up to twice as long as that for boosting. For reasons of brevity, the results for the bagging estimator were not reported in the paper.

## Code Availability

Custom code using R open-source software and packages (R Core Team, 2022).
